# Piezoelectric Signals in Vascularized Bone Regeneration

**DOI:** 10.3390/biom11111731

**Published:** 2021-11-20

**Authors:** Delfo D’Alessandro, Claudio Ricci, Mario Milazzo, Giovanna Strangis, Francesca Forli, Gabriele Buda, Mario Petrini, Stefano Berrettini, Mohammed Jasim Uddin, Serena Danti, Paolo Parchi

**Affiliations:** 1 Department of Surgical, Medical, Molecular Pathology and Emergency Medicine, University of Pisa, 56126 Pisa, Italy; delfo.dalessandro@unipi.it (D.D.); francesca.forli@unipi.it (F.F.); s.berrettini@med.unipi.it (S.B.); 2 Department of Translational Research and of New Technologies in Medicine and Surgery, University of Pisa, 56126 Pisa, Italy; claudio.ricci@med.unipi.it (C.R.); paolo.parchi@unipi.it (P.P.); 3 The BioRobotics Intitute, Scuola Superiore Sant’Anna, 56024 Pontedera, Italy; mario.milazzo@santannapisa.it; 4 Department of Civil and Industrial Engineering, University of Pisa, 56122 Pisa, Italy; g.strangis@studenti.unipi.it; 5 Department of Clinical and Experimental Medicine, University of Pisa, 56126 Pisa, Italy; gabriele.buda@unipi.it (G.B.); mario.petrini@med.unipi.it (M.P.); 6 Department of Chemistry, University of Texas Rio Grande Valley, Edinburg, TX 78539, USA; mohammed.uddin@utrgv.edu

**Keywords:** biomaterials, scaffold, tissue engineering, angiogenesis, osteogenesis, stem cells, mesodermal progenitor cells, orthopedics, otology

## Abstract

The demand for bone substitutes is increasing in Western countries. Bone graft substitutes aim to provide reconstructive surgeons with off-the-shelf alternatives to the natural bone taken from humans or animal species. Under the tissue engineering paradigm, biomaterial scaffolds can be designed by incorporating bone stem cells to decrease the disadvantages of traditional tissue grafts. However, the effective clinical application of tissue-engineered bone is limited by insufficient neovascularization. As bone is a highly vascularized tissue, new strategies to promote both osteogenesis and vasculogenesis within the scaffolds need to be considered for a successful regeneration. It has been demonstrated that bone and blood vases are piezoelectric, namely, electric signals are locally produced upon mechanical stimulation of these tissues. The specific effects of electric charge generation on different cells are not fully understood, but a substantial amount of evidence has suggested their functional and physiological roles. This review summarizes the special contribution of piezoelectricity as a stimulatory signal for bone and vascular tissue regeneration, including osteogenesis, angiogenesis, vascular repair, and tissue engineering, by considering different stem cell sources entailed with osteogenic and angiogenic potential, aimed at collecting the key findings that may enable the development of successful vascularized bone replacements useful in orthopedic and otologic surgery.

## 1. Introduction

Stem cells are the foundation of tissue development and regeneration. Since tissues develop as three dimensional (3D) structures, tissue engineering has emerged in recent decades as a multidisciplinary field to enable 3D regeneration [1]. As such, it is based on three pillars, namely, cells (primarily, stem cells), biomaterial scaffolds (to allow 3D spatial organization of the cells), and stimulatory factors (to carry out fundamental cellular functions, such as proliferation and/or differentiation). Under a classical approach, tissue engineering provides cells with differentiation potential to be seeded on biocompatible scaffolds; the resulting cell/scaffold constructs are cultured in media containing chemical factors stimulating cell proliferation and/or differentiation to mimic in vitro the native microenvironment of a specific anatomical district [2]. In this view, the biomaterial scaffold provides a temporary artificial extra cellular matrix (ECM) to allow the neo-tissue to grow in 3D. In recent years, the scientific research has highlighted the role of other stimulatory factors, e.g., exerted by the surrounding fluidic microenvironment, or by the scaffolds themselves, such as mechanical and architectural [3]. Exogenous electric signals can also be applied via biomaterials interacting with cells and have resulted to be capable of affecting cell function [4].

**Figure 1:**
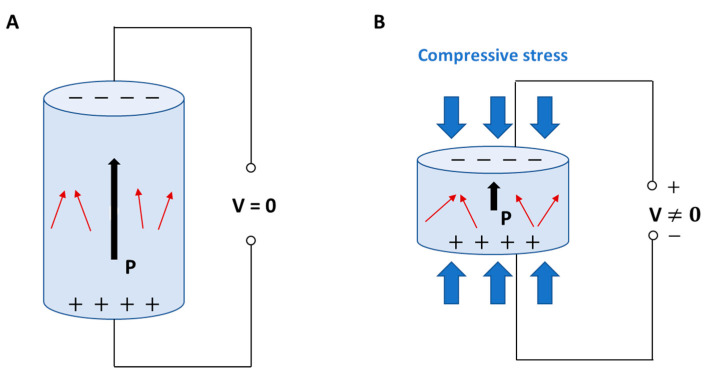
Direct piezoelectric effect in a biomaterial element: (**A**) without external stress, and (**B**) subject to compressive stress with charge generation. P: polarization vector; V: voltage; red arrows: dipoles; black arrows: direction of polarization vector.

Recently, the role played by the different distribution of electrical charges in biological systems has been investigated, identifying it as a further pre-eminent biological stimulus able to modify and guide some biological events, as well as chemical stimulating factors [5]. Indeed, bioelectricity is a fundamental characteristic of organisms, including humans. The best known electrically stimulable tissues, including nerve and muscle tissues, are made up by cells that depolarize their membrane by generating an electric potential. Electric signals can regulate physiological events, such as the muscle contraction and the voluntary and involuntary functions managed by neurons. Electric stimuli can be induced directly, and indirectly as a consequence of magnetisms or mechanical forces [6]. In particular, the piezoelectric effect is the ability of certain materials to generate an electric charge differential in response to an applied mechanical stress and vice versa (Figure 1) [7].

**Figure 2:**
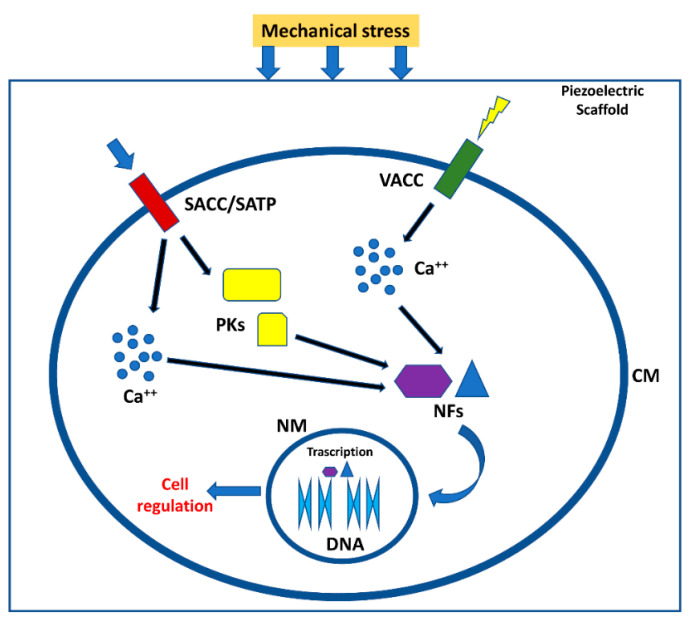
Effect of piezoelectric biomaterials on the cells. Mechanical stress compresses the scaffold or ECM generating the activation of voltage-dependent channels and channel/transmembrane proteins activated by mechanical stimuli. Calcium ions and protein kinases generate a signal cascade that in turn activate nuclear factors able to migrate in cell nuclei, generating different cell responses [4]. SACC = stretch activated calcium channels; SATP = stretch activated transmembrane proteins; VACC = Voltage activated calcium channels; PKs = protein kinases; NFs = nuclear factors; CM = cell membrane; NM = nuclear membrane.

When a dielectric solid is placed in an externally applied electric field, the relative positions of both nuclei and electrons change, generating electric dipoles that determine, in turn, the material polarization. Dielectrics are poor/non-electrically conductive materials, but susceptible to polarization in the presence of an electric field, thus behaving as capacitors. Piezoelectric materials are a subset of dielectric materials that can be polarized through an externally applied mechanical stress [7]. Piezoelectricity is determined by the characteristics of some chemical crystalline structures, namely, non-centrosymmetric. When the charge balance, overall neutral in rest condition, is disturbed by an external stress applied to the crystalline network, small dipoles are created and material surfaces result to be charged with opposite polarity during the time of mechanical force application [8,9,10]. The piezoelectric effect has been recognized in highly crystalline structures, such as perovskite-like ceramics, and to a lower extent also in some polymers and biological materials provided with specific crystalline phases, where it mainly derives from the orientation of the polymeric crystallites and their intrinsic piezoelectric properties [11]. In the live matter, piezoelectricity is particularly significant in some biological materials, such as wood, as well as in tissues, such as tendon and bone, among others [12]. In bone, piezoelectric stimuli enabled by ECM in response to a mechanical stress or strain can influence the remodeling of osseous tissue or modulate the production of coagulation factors by the endothelium layering the internal walls of blood vessels [8]. Moreover, piezoelectric stimuli can regulate cell cycle, migration, proliferation, and differentiation (Figure 2) [13,14,15].

Interestingly, piezoelectricity has been found to be incredibly widespread in the body tissues, being ubiquitously present in some ECM molecules, such as collagen and elastin [16,17]. Therefore, it is very important that polymeric biomaterials employed for the fabrication of tissue engineering scaffolds exhibit this property. Indeed, piezoelectric biomaterials have been investigated in several tissue engineering studies aimed at regenerating osseous [18,19], muscular [20], neural [21] and cardiovascular tissues [22], as well as at healing wounds [23], and repairing vascular tissue [24].

It is expected that the application of piezoelectric biomaterials can guide tissue regenerative processes led by stem cells in a biomimetic fashion. Indeed, these materials, because of mechanical signals due to cell attachment or mechanical stress, generate variations in electrical potential without the need for power supply devices. Obviously, these materials must be biocompatible and have sufficient piezoelectric properties for the generated electric charges to be properly sensed by the cells [25,26]. Piezoelectric biomaterials account for piezoelectric polymers, piezoelectric ceramics and their composites [27]. In particular, polymer-based materials, including nanoceramic/polymer composites, possess the advantage of processing flexibility, which makes them appealing for diverse manufacturing technologies, including 3D printing and electrospinning [25,28]. Since pure ceramics show the strongest piezoelectricity but are mechanically hard and fragile materials, difficult to process, the use of piezoelectric biomaterials for scaffolding mostly relies on ceramic/polymer composites, which combine the advantages of both [28]. The structural requirements of piezoelectric polymers include the presence of permanent molecular dipoles, the ability to align or orient molecular dipoles and to sustain their alignment once achieved, along with capability of the material to support suitable mechanical stress [29]. Finally, the polymers entitled with the highest piezoelectric properties, i.e., belonging to the poly(vinylidene fluoride) (PVDF) family, are chemically stable. Therefore, the lack of biodegradable polymers showing relevant piezoelectricity is still a bottleneck of tissue engineering applications, which is invoked to be possibly overcome by polymer nanocomposites.

This review summarizes the special contribution of piezoelectricity for bone and vascular tissue regeneration, including osteogenesis, angiogenesis, vascular repair, and tissue engineering, by considering different stem cell sources entitled with osteogenic and angiogenic potential, aimed at collecting the key findings that may enable the success of novel smart vascularized bone replacements in orthopedic and otologic surgery.

## 2. Vascularized Bone as a “Piezoelectric” Regenerative Target

**Figure 3:**
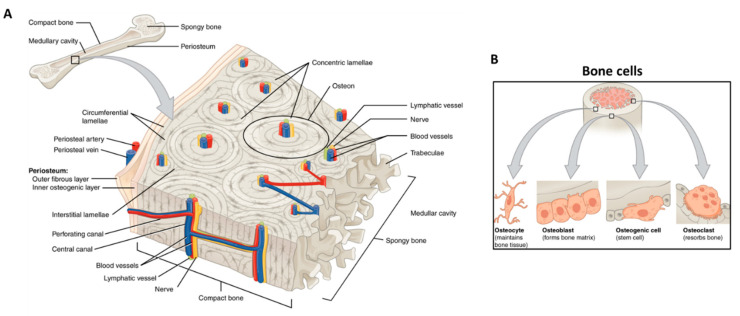
Bone tissue schematic showing: (**A**) bone structures (e.g., osteon, blood vessels, lamellae, periosteum, and trabeculae); and (**B**) the main bone cells (i.e., osteocyte, osteoblast, osteogenic cell, osteoclast) and their location. Adapted from OpenStax.

As a part of the vertebral skeleton, bone is mineralized tissue entailed with mechanically supportive, protective, movement, metabolic, remodeling, and hemopoietic functions [30]. Histologically, the osseous tissue is constituted by a cellular component, immersed in a hard ECM, which is predominantly formed by mineral salts (~70% *w*/*w*), collagenic proteins (~20% *w*/*w*), and, to a lesser extent, amorphous matter (~2% *w*/*w*). The complement (8% *w*/*w*) is water, which improves bone ductility [31,32]. Collagen type I is the most represented collagenous protein of bone, while the amorphous matter mainly consists of unsulfated glycosaminoglycans [30]. The mineralization process occurs via precipitation of hydroxyapatite [Ca_10_(PO_4_)_6_(OH)_2_] by the surrounding extracellular liquids on pre-osseous collagen matrix as a nucleating substrate. Bone tissue exists in two main subtypes: cortical (or compact) on the outer surfaces, and cancellous (also known as spongy, or trabecular) in the interior [33]. The structural unit of compact bone is the osteon, or Haversian system, which consists of a central canal serving as a passageway for blood vessels and nerves, surrounded by tubular subunits of bone ECM, namely, the bone lamellae [34]. On the other hand, Volkmann’s canals are other vascular channels that run perpendicular to the major axis of the osteons (Figure 3A). While compact bone is just provided with incorporated vessels, the spongy bone consists of trabeculae perfused by vasculature and bone marrow (BM), the latter representing the hematopoietic cell factory [35,36].

The bone cells, representing a low volumetric fraction of the tissue, are responsible for the fine and fundamental control and maintenance of bone homeostasis. Three main types of cells concur with this mechanism: the osteoblasts (or bone building cells), the osteocytes, and the osteoclasts (or bone destroying cells) (Figure 3B) [33].

In time, the osteoblasts are found to be enclosed inside the matrix they have built, thus becoming osteocytes. These cells do not divide and have an average lifespan of about 25 years. Moreover, they can reversibly turn back into osteoblasts, and live a new life as bone constructors [37]. The osteocytes are able to interact with other cells, forming a communication network with osteoprogenitor cells, osteoblasts, and osteocytes where the osteocytes act as mechanosensors [33]. They are stellate cells, with a biconvex-shaped cellular body and numerous cytoplasmic extensions. As such, between the plasma membrane of the cell body and the extensions, the mineralized matrix remains a thin space occupied by osteoid tissue [34]. On bony surfaces, the osteoblasts are responsible for the synthesis and mineralization of the matrix; in addition, they are joined together with the neighboring osteocytes through gap junctions, through which the cells exchange signal molecules for the coordination of the metabolism and deposition of the bone matrix. Osteoblasts secrete growth factors, including transforming growth factor beta (TGF-β), which acts in an autocrine and paracrine manner, modulates the proliferation of osteoprogenitor cells, promotes their differentiation, increases the metabolism of mature osteoblasts, and is sensitive to electric stimulation [38,39]. These cells are able to respond to various stimuli, including mechanical, intervening again in growth and remodeling [40]. Remarkably, the vascularization process is crucial in bone regeneration as well as in remodeling and homeostasis, and the complex pathways that lead to angiogenesis and osteogenesis are interdependent [41]. In such a complex tissue microenvironment, mechanical stimuli are finely sensed by osteocytes and act as signals for bone cells to modify their gene expression and finally induce synthesis or degradation of the bone ECM. Differently from the osteoblasts/osteocytes, of mesenchymal origin, the osteoclasts are polynucleated giant cells, belonging to the macrophage family (i.e., of hematopoietic origin) [42]. The articulated system of microscopic channels and lacunae in the compact bone, and/or cavities in the spongy bone permits an efficient cellular cross-talk that enables the complex mechanism of bone growth and remodeling. By virtue of remodeling, the osseous tissue is also able to optimize its shape and resistant sections depending on the load to bear, thus showing astonishing adaptive changes [43].

**Figure 4:**
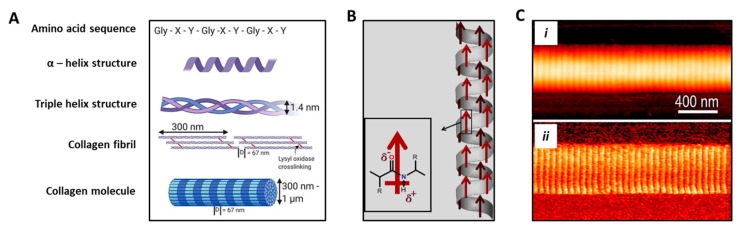
Structure and piezoelectric behavior of collagen molecule. (**A**) Hierarchical schematic showing the amino acid sequence in which X and Y are usually proline and hydroxyproline, thus being able to form a unique α helix secondary structure. Fibrillar collagen is a triple helix containing crosslinks formed through the action of lysyl oxidase. Collagen fibrils form fibers with varying thickness and a D-banding pattern of 67 nm. Adapted from [47], reused under Creative Commons Attribution (CC BY) license. (**B**) Schematic showing permanent polarization in α-helix. Red arrows indicate the direction of the dipole moment. Adapted from [26], reused under Elsevier & Copyright Clearance Center (license number 5185310303078). (**C**) Single collagen fibril analysis obtained via atomic force microscopy: (***i***) topography, and (***ii***) corresponding shear piezoelectricity obtained under piezoforce microscopy mode. Reprinted with permission from [50], Copyright 2009, American Chemical Society.

Bone has also been recognized to show piezoelectricity, which is suggestive of important signaling involved in tissue function. Collagen fibers are considered to play a role in bone piezoelectricity [44]. The collagen molecule is made up by three polypeptide strands, connected by hydrogen bonds and twisted to form a triple helix structure. As such, the it can behave as a crystal, which produces the piezoelectric effect by orientation of dipoles involving NH and CO groups [12]. Collagen is produced by a fibrillogenic process in which the procollagen chains produced at an intracellular level nucleate at one end to give rise to a procollagen trimer, which undergo a self-assembly process occurring extracellularly. In bone, collagen type I accounts for 90% of collagenic proteins. In osteoblasts, COL1A1 gene produces the pro-α1(I) chain [38,45]. This chain combines with another pro-α1(I) chain and with a pro-α2(I) chain (produced by the COL1A2 gene) to make a molecule of type I procollagen. These triple-stranded, rope-like procollagen molecules are extruded out of the cell and further processed by enzymes to arrange themselves into long, thin fibrils that cross-link to one another in the inter-cellular spaces [46]. The cross-links result in the formation of very strong mature type I collagen fibers (Figure 4A) [47,48]. Collagens are the main constituents of bone as well as vascular tissues. All vessel lumens are made of endothelial cells (ECs) anchored on an underlying basement membrane, a thin structure made of laminin, collagens type IV, type XV, and type XVIII, among other biomolecules. Under the basement membrane, normal vessels contain elastic fibers and collagens with different amounts, depending on whether they are arteries or veins, including fibrillar collagens type I and III [49].

Collagen fibrils show polarization along their axial direction (Figure 4B,C). The piezoelectric effect is given by the imperfect hexagonal symmetry of their structure at the nanometric level. In addition, the generation of electrical potentials in bone undergoing mechanical stress is determined by ion movements in the mineralized matrix [50,51]. The piezoelectric action can in turn alter the chemistry of the collagen molecules or affect the cellular activity responsible for the control mechanism involved in the bone growth and remodeling [52].

More recently, it has been shown that synthetic nanocrystalline hydroxyapatite films exhibit strong piezoelectricity [53]. In particular, in 2005 two polar symmetries for hydroxyapatite were proposed, i.e., monoclinic and a hexagonal, which are not centrosymmetric, thus opening up new scenarios for hydroxyapatite contribution to bone piezoelectricity [54]. The small energy difference between these polar symmetries and the non-polar centrosymmetric counterpart, indicates that nanosized hydroxyapatite may have these polar structures stable as a consequence of large surface energy. For this reason, it is possible that not only collagen fibrils, but also hydroxyapatite, produced by biomineralization process in the form of nucleated and precipitated nanocrystals, concur to make bone a piezoelectric material [55]. In 1969, elastin was entailed (along with collagen) as piezoelectric constitutive matter of large blood vessel walls, led by preferred anisotropic orientation of the tissue structure [56]. Elastin fibers are at the basis of blood vessel rheological properties, such as the post-systolic elastic recoil [57]. Elastin has recently been discovered to demonstrate intrinsic polarization at the monomer level, thus to be considered analogously to a classical perovskite unit cell [16]. The diffused evidence of piezoelectricity in bone as a vascularized tissue is thus highly supportive for including piezoelectric stimuli to attain its functional regeneration.

## 3. Cell Sources Used to Engineer Vascularized Bone Substitutes, and Cellular Susceptivity to (Piezo)electric Stimuli

**Figure 5:**
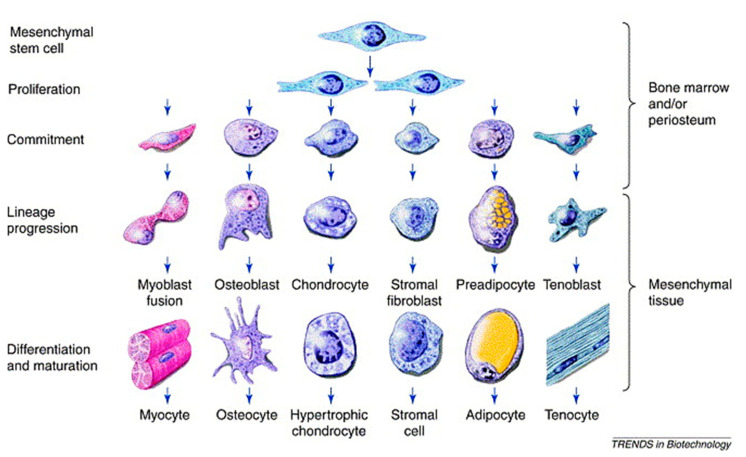
Schematic of the mesengenic process showing MSCs upstream and their differentiation capacity across diverse mesoderm tissues, including bone. Reprinted with permission from [61], under Elsevier and Copyright Clearance Center (license number 5166410951759).

The mesenchymal stem (or stromal) cell (MSC) is considered to be upstream the osteogenic lineage, and COL1 gene is an indisputable early marker of osteogenesis [45,58,59]. However, the ancestor progenitor of the MSC is still a subject of debate, the pericyte being a possible candidate according to some hypotheses [59,60]. The mesengenic process describes the descendances of the MSC family tree, which includes bone, cartilage and vasculature, among other mesodermal origin tissues (Figure 5) [58]. In the osteogenic lineage, various progenitors at diverse differentiation stages have been described, which all share the osteogenic hallmark, namely, core binding factor alpha-1 (CBFA1) known also as Runx2 [45]. Among them, osteoprogenitors are found in the inner layer of the periosteum, called the osteogenic layer of Ollier, and in the endosteum, resemble MSCs and can differentiate into osteoblastic cells also exploiting bone morphogenetic proteins (BMPs).

The choice of the most suitable cell type for tissue regeneration studies is fundamental to obtain a functional tissue-engineered construct. To obtain a vascularized bone substitute, cell precursors able to differentiate into bone cells (i.e., osteoblasts), as well as precursors regenerating the vascular endothelium and promoting angiogenesis, are considered. Primary osteoblasts are certainly the most immediate cell type considered for tissue engineering studies applied to bone tissue regeneration, However, they need to be isolated from bone biopsies and the long time for their isolation and expansion, along with their low viability, make primary osteoblasts poorly useful for developing customized 3D models [62,63].

An interesting and widely studied alternative is represented by MSCs, isolated for the first time from BM, but also present in many other anatomical sites, such as muscle and adipose tissue, dental pulp, and umbilical cord blood, among others [64,65,66]. BM-derived MSCs represent 0.10–0.01% of the entire cellular population present in the BM. They are able to adhere to the culture flask and show a spindle shape similar to that of fibroblasts. Moreover, MSCs are characterized by the absence of hematopoietic markers, such as CD34, CD45, CD14, and by the expression of a specific pattern of adhesion molecules, such as CD90, CD105, and CD44. They are able to differentiate towards the adipogenic, osteogenic, and chondrogenic lineages, and produce a variety of cytokines, which, in the preclinical model, favor engraftment and reduce the rejection of transplants [67,68,69]. MSC isolation from BM, however, is somehow painful for the patient, thus other anatomical sites could be considered.

Adipose tissue-derived MSCs (AD-MSCs) are easily isolated from lipoaspirates in larger amount than MSCs isolated from BM [70,71]. They show features similar to the BM-MSCs, but show an increased differentiation capability towards adipogenic lineage and a decreased potential towards the osteogenic lineage [72]. In addition, AT-MSCs highlighted in vivo ability to secrete proangiogenic mediators and molecules stimulating the activity of the bone tissue [73].

**Figure 6:**
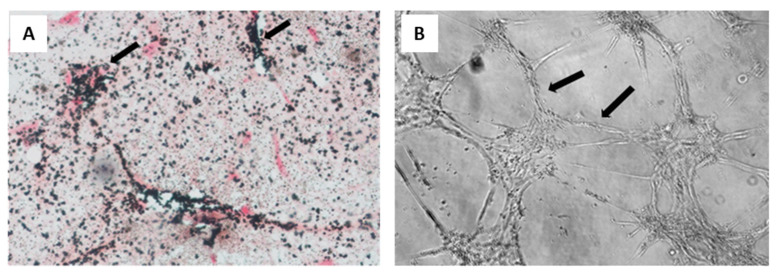
Osteogenic and vasculogenic potential of dental pulp MSCs: (**A**) von Kossa staining of osteo-differentiated dental pulp MSCs, showing calcium deposits in black and cells in red. Arrows point to representative areas of intense mineral deposition in proximity to osteoblasts; and (**B**) light micrograph of dental pulp MSCs after endothelial differentiation, showing capillary tube-like structures. Reprinted with permission and adapted from [61], under Elsevier and Copyright Clearance Center (license number 5166500137992).

Oral tissue is a good source of stem cells, thus being considered a valuable tool for bioengineering [74]. They can be isolated from various sites, such as the periodontal ligaments, the apical papilla, and exfoliated temporal teeth [75]. These MSCs are similar to BM-MSCs: they adhere in culture flasks and show fibroblastic-like morphology; moreover, they show stem cell markers and hematopoietic markers are not present; finally, they are able to differentiate with towards adipogenic, chondrogenic, and osteogenic lineages in a similar fashion to BM-MSCs [76,77]. In addition to stimulating bone formation in vivo and in vitro, these cells are able to promote angiogenesis (Figure 6) [78].

Another cellular source that has been considered to obtain 3D models for bone regeneration is represented by induced pluripotent stem cells (iPSCs). IPSCs are obtained by genetic engineering procedures, transferring transcription factors such as Oct4, Sox2, Klf4, and c-Myc to human primary cells, making them acquire pluripotency characteristics similar to those of embryonic cells. Therefore, such cells can also differentiate into osteoblasts and osteoclasts. However, the complex procedure required to obtain iPSCs and the low efficiency of the procedure render them not yet suitable to obtain predictive preclinical models [79,80,81].

To achieve endothelial regeneration for a correct bone vascularization, it is necessary that the endothelial cells (EC) already present in a vessel migrate and/or that endothelial progenitor cells (EPCs) deriving from BM are recruited to the lesion site [82,83]. EPCs are a population of unipotent progenitors with self-renewal, clonogenicity and differentiation capability present in the peripheral blood of many organs such as spleen, umbilical cord, liver, kidney [84,85,86]. Recent studies indicate that EPCs can promote endothelial regeneration not directly, but through the release of soluble factors such as bone morphogenetic proteins (BMPs), vascular endothelial growth factor (VEGF), TGF-β, and by recruiting resident MSCs and ECs at the bone formation site [87,88,89,90].

**Figure 7:**
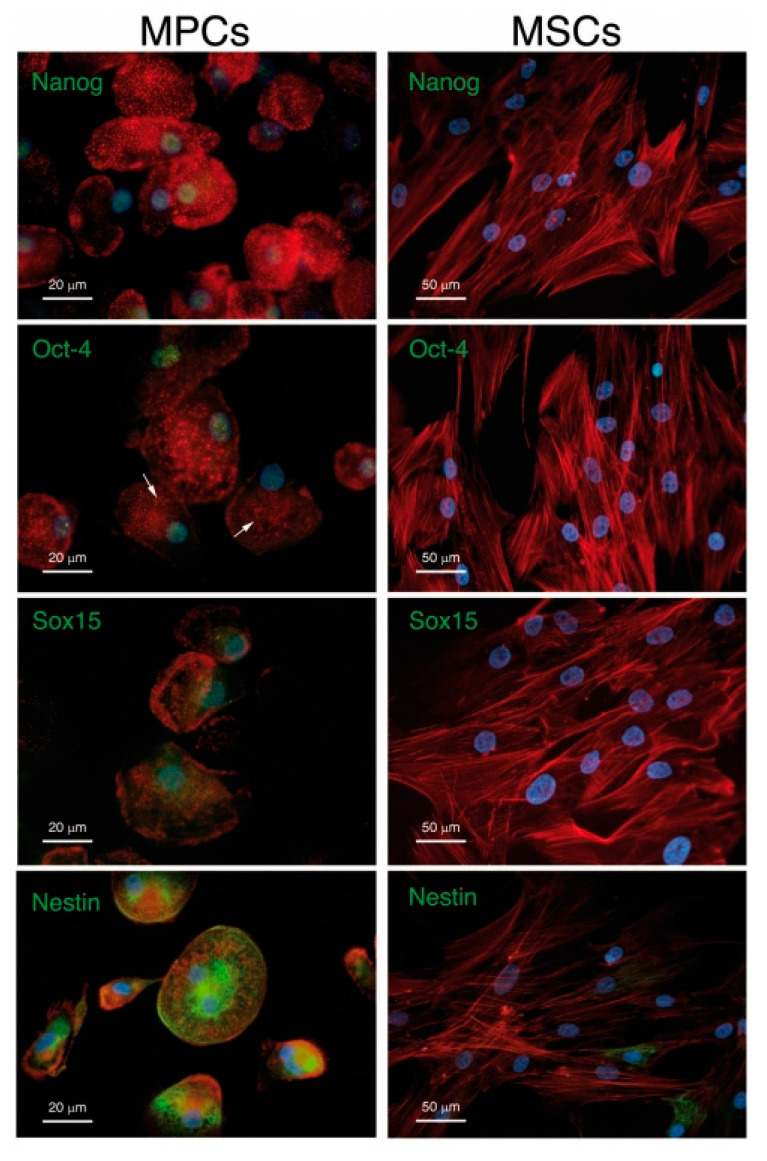
MPCs versus MSCs. Immunofluorescent staining confirms the expression of Nanog, Oct-4 and Sox15 (green) in MPC but not in MSC nuclei (blue) MPCs and MSCs show different spatial organization of F-actin (red). MPCs also differ from MSCs due to their unexpected high expression of well-organized Nestin filaments (green). Reused from [89] under Creative Commons Attribution License.

Mesangiogenic (or mesodermal) progenitor cells (MPCs), described in 2008 as identified in human BM mononuclear cell cultures during isolation and expansion of MSCs under animal-free conditions, are a powerful cell source to be considered to the endothelial and bone regeneration (Figure 7) [91]. An extensive phenotypic and functional characterization of these interesting cells showed a distinct phenotype from MSCs and the expression of CD45, although at much lower levels of leukocytes. Furthermore, the gene expression profile of MPCs revealed pluripotency markers, such as Oct-4, Nanog and Nestin (Figure 7) [92,93]. Rigorous studies have demonstrated a differentiative capability of MPCs towards MSCs, which in turn can differentiate in vitro and in vivo towards osteoblastic, chondrogenic, and adipogenic lineages, among others [94]. Furthermore, MPCs retained a demonstrated angiogenic potential both in vitro and in vivo, which is lost upon differentiation towards the mesenchymal lineage [95].

Efforts to determine which BM sub-population might be capable of generating MPCs in culture have led to the identification of a single subpopulation with monoblast-like characteristics, called Pop#8, which showed high potential for endothelium regeneration [96].

**Figure 8:**
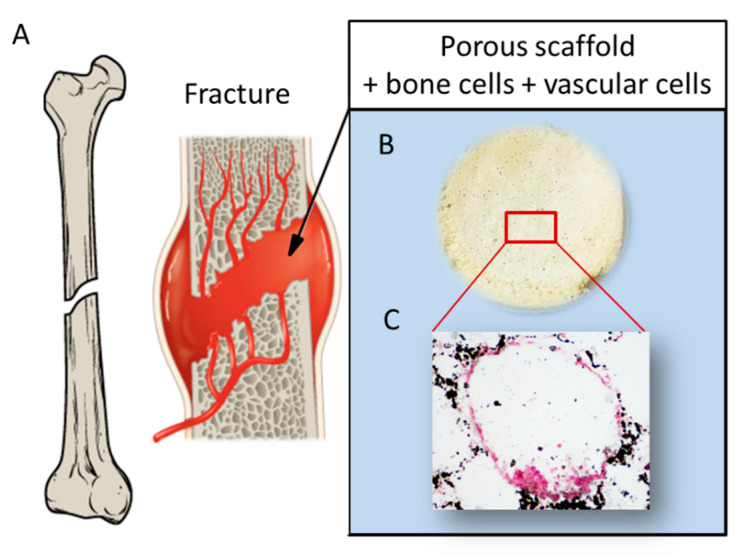
Schematic showing: (**A**) a fracture involving bone tissue including vasculature; (**B**) a porous scaffold; and (**C**) histological analysis displaying a pore colonized by a dual cell population: osteoblast-like cells producing mineral matrix (by von Kossa staining positive, in black), and endothelial cells surrounding the pore walls (von Kossa staining negative, in red), reprinted with permission and adapted from [101], under John Wiley and Sons Copyright Clearance Center (license number 5170741359741).

Remarkably, MPCs, as a single stem cell source, retain the potential to generate all the populations necessary for prevascularized bone substitutes under a tissue engineering approach [97]. For these reasons, co-culture of MPC-derived EPCs and MPC-derived MSCs on a biocompatible and bioresorbable scaffold is expected to give rise to a pre-vascularized, fully-autologous, and functional bone construct relevant for clinical applications. Simultaneous employment of different cell populations could improve the efficiency of the regenerative process. Co-cultures of MSCs and endothelial precursor cells have demonstrated that these different cell populations have a synergistic action. Indeed, studies where MSCs and EPCs or human umbilical vein cells (HUVECs) were co-cultured on 3D scaffolds have shown an improvement in the survival rate of MSCs and stimulation of bone differentiation as well as angiogenesis [98,99,100,101]. A schematic of MSC and HUVEC co-culture on a scaffold is reported in Figure 8. Recent studies have highlighted the absence of MPCs in the adipose tissue, thus raising doubt about the capability of AD-MSCs to promote an efficient osteogenesis, which includes vascularization potential [96].

Bioelectricity, which originates from transmembrane voltage gradients at a cell level, is known to play a central role in orchestrating cell function during embryonic development, as well as in tissue regeneration and repair, thus strongly involving stem cells [102].

Human MSCs treated with exogenously applied electrical stimulation (ES) at 15 V for 10 min every day for 4 weeks through parallel plate electrodes, showed a significant upregulation of osteocalcin and alkaline phosphatase (ALP) gene expression, as well as calcium deposition, possibly as a consequence of augmented cytosolic Ca^2+^ ion flux [103]. ES has also demonstrated to strongly affect, in diverse ways, cell alignment according to the electric field vector direction. As an example, cardiac and endothelial progenitor cells, vascular ECs, MSCs and adipose-derived stromal cells aligned perpendicular to the direction of the electric field vectors to minimize the field gradient across the cell, whereas ventricular and cardiac myocytes, myoblasts, and osteoblasts aligned parallel to the field vectors since ES induced cell cytoskeleton rearrangement [104]. Upon ES application, both MSCs and ECs, independently cultured, oriented perpendicular to the field vectors; interestingly, EC morphology resembled that of the inner layer of the blood vessel, with consistent perpendicular alignment to the field vector, which was suggestive of high angiogenic potential [105,106]. As an exogenous stimulus, ES performed at an intensity lower than 2 V∙cm^−1^ for 14–28 days was able to induce MSCs towards osteogenesis (in place of chondrogenesis), still in presence of dexamethasone [107]. Such evidence highlights the prominent role of electric signals in stem cell differentiation, which appears to be specifically important in osteogenesis and vasculogenesis. Piezoelectricity imparts mechanically-driven ES based on polarization effects; thus, it is mediated by mechanically solicitated native ECM components, or biomaterials, usually located out of the cells. Hence, piezoelectricity is usually relevant where mechanical loads are predominant. The intensity, duration and type of mechanical stimulation, the specific piezoelectric material, and the resulting electric output thus concur with the effective ES perceived by the stem cells with activation of their differentiative pathways. In fact, it has been reported that MSCs seeded on quartz glasses and administrated with ultrasound as a mechanical stimulation, in the range of 1–20 mW∙cm^−2^ intensity, resulted in the activation of a chondrogenic differentiative route [108]. In a comparative study using poled and non-poled electrospun PVDF scaffolds as substrates, it was demonstrated that human MSCs selected either osteogenic or chondrogenic lineage differentiation depending on the entity of the output voltage (or streaming potential) generated by the piezoelectric scaffold, namely, either high (i.e., 61.1 ± 1.5 μV) or low (25.2 ± 2.5 μV) voltage, respectively, upon dynamic sinusoidal compression at 1 Hz and a deformation of 10% obtained using a bioreactor [109]. Such electric potentials are lower than those applied by direct ES; however, in piezoelectric stimulation, the cells sense the mechanical stress in addition, and putatively synergistically, to the generated ES. In fact, pure mechanical stimuli, including substrate stiffness and external loads, which may be applied in vitro by bioreactors, have largely been demonstrated to influence stem cell fate [110]. 

## 4. Piezoelectricity in Vascular Grafts and Endothelial Regeneration

**Figure 9:**
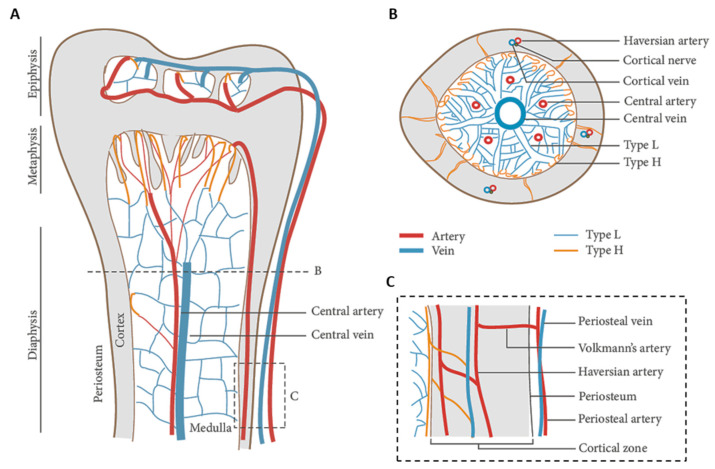
Blood vessel arrangement in long bones: (**A**) longitudinal view showing arteries branching into H type capillaries, and veins branching into L type capillaries in the epiphysis, metaphysis, and diaphysis; (**B**) cross view showing a main central vein and a few arteries in the medullary region; and (**C**) zoomed-in panel showing the connection between cortical and medullary blood flow. Adapted from [111] and reused under Creative Commons Attribution (CC BY) license.

Blood vessels are an integral part of the skeletal system and essential in maintaining bone homeostasis. The spatial arrangement of the articulated vascular network in bone enables the optimal delivery of oxygen and metabolites, as well as carbon dioxide and catabolite removal within the BM compartment (Figure 9) [111].

Blood supply to bone is provided by arteries entering the cortical region, and a central artery, which divides into arterioles and capillaries. Exhausted blood is finally collected into a main central vain and thus returns to pulmonary circulation. Regenerating vascularized bone is therefore impellent for gathering clinical success. In fact, resection of bone tumors, bone cysts or trauma result in extended skeletal defects that need to be filled using large volumes of substitutive material, which makes neo-vascularization fundamental for bone cell survival. As a consequence of the inflammatory process taking place after surgery, new vessels are only transiently being formed deriving from the neighboring vasculature, and even in case of porous scaffolds, the process of vasculature infiltration is usually too long, i.e., lower than 1 mm per day [112]. Among several other strategies, which will be discussed in Section 5, vascularized bone grafts can be obtained by combining bone tissue engineering with microsurgery, for example, by implantation of an arteriovenous loop around the construct [113]. Under a clinical perspective, reconstructing vasculature at the moment of large bone defect reduction is highly relevant, which can be performed, if possible, by rearranging existing vasculature, or even putatively using vascular grafts.

Artificial vessels have been developed for repairing the vascular system, thus are not directly related to bone regeneration. However, due to the fundamental nature of vasculature in bones, in this review we revise the current knowledge about piezoelectric materials for vascular monitoring, repair, and regeneration, independently of bone engineering, with the objective of providing interesting knowledge to develop new materials able to promote both bone and vascular regeneration by availing themselves of piezoelectricity. A vascular graft is an artificial duct drawn on the patient’s artery that is used to bypass a damaged vessel. Atherosclerosis is one of the pathologies responsible for a damage to the vascular system, which behaves as a progressive disease characterized by the accumulation of lipids in the blood vessels [114]. The ischemic events resulting from the disease often require the replacement of some part of a vessel. In many cases, autologous tissue is used; however, the limited availability of the material, the multiple surgical interventions required, the morbidity of the donor site, and a high failure rate, make this a not a very functional technique, prompting scientific research to identify other reparative strategies [115]. The failure of the vascular graft is not identifiable by the presence of symptoms, but consists of an inefficient blood flow leading to thrombus formation. To prevent the problem, it is necessary to constantly monitor the implant, because intervening at late stages can be difficult, leading to the patient’s death [116]. Currently, techniques are employed that involve the use of ultrasounds, computed tomography, and angiograms to define blood pressure and flow velocity in the lumen of the vascular graft [117,118]. However, these procedures are complex, expensive, and can be toxic to the patient [119,120]. New solutions to monitor the grafts consist of the use of appropriate sensors, which, placed in direct contact with the blood flow, allow the blood pressure to be detected at the implant level. However, they alter the structure of the vessel and can cause turbulence [117]. To overcome this problem, sensors consisting of membranes were mounted on the external wall of a graft constituted by polydimethylsiloxane (PDMS), a chemically inert elastomer, thermally stable, easy to handle and to shape, and interesting for biomedical applications [121]. They are able to measure blood pressure indirectly and record the mechanical stresses of the vascular wall because of the blood flow passage [122].

Piezoelectric materials have proved to be very useful in providing a rapid response to changes in vessel pressure. Indeed, they are very flexible and sensitive to mechanical stimuli, which makes them perfect for this type of application [123,124,125]. However, piezoelectric sensors are also bulky, and this alters the mechanical response of the vascular wall. For this purpose, a very thin piezoelectric sensor of aluminum nitride integrated with the prosthesis has been developed, which showed to be non-toxic and very functional [126]. Alternatively, piezoelectric sensors based on nanoceramic zinc oxide (ZnO) and lead zirconate titanate (PZT) were created, but issues concerning biocompatibility and fragility have occurred [127].

A different approach is to create synthetic substitutes instead of the autologous graft, with the advantage to have a wide availability of easily customizable material. The synthetic substitutes are based on biocompatible materials commercially in use for a long time, such as poly(tetrafluoroethylene) and poly(ethylene terephthalate), but they are used exclusively to replace vessels with a diameter greater than 6 mm, as their application in smaller diameter replacements has caused scarce reendothelialization with consequent thrombogenesis [128]. An interesting alternative to overcome this limitation is represented by tissue engineered vascular graft (TEVGs), based on materials that must meet three essential requirements: they must have mechanical properties matching those of the blood vessels, high biocompatibility, and adequate porosity to favor a correct reendothelialization, the latter being a fundamental aspect due to the endothelium playing an active role in all physiological processes, such as homeostasis and the regulation of vascular tone. Elastomers are a class of materials with high elasticity that can be finely adjusted. Polyurethanes are flexible and biocompatible as well as have a high tensile strength. They can also combine with PDMS to increase elastic properties [129,130]. Vascular grafts have been developed containing silver nanoparticles and carbon nanotubes exhibiting antithrombotic and antibacterial properties [131,132].

**Figure 10:**
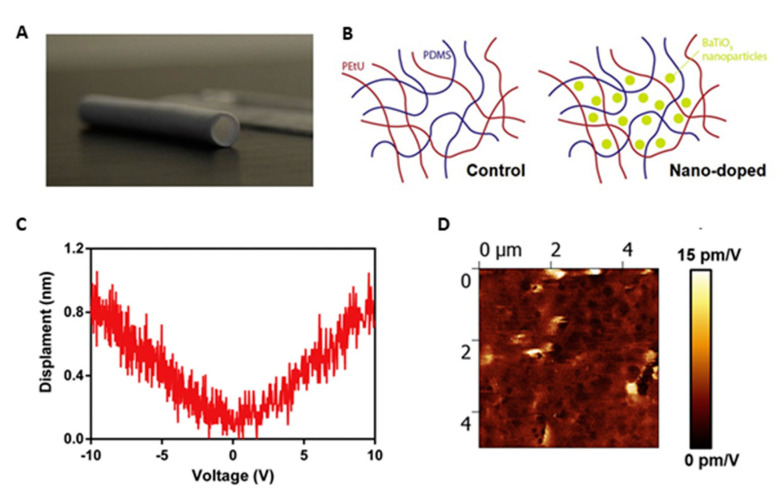
Piezoelectric small caliber vascular graft: (**A**) photograph; (**B**) schematic of the polymer network incorporating BaTiO_2_ nanoparticles; (**C**) representative displacement-voltage curves, obtained for the nano-doped samples; and (**D**) piezoelectric force microscope images showing signal amplitude maps and corresponding average and maximum d_33_ values for the nano-doped samples. Reprinted with permission and adapted from [61], under Elsevier and Copyright Clearance Center (license number 5166600438237).

Since the vessels retain piezoelectric properties, mainly attributable to collagen and elastin present in ECM [133,134], piezoelectric polymers can be considered in view of promoting vascular regeneration [25]. PVDF, often used in association with trifluoroethylene, P(VDF-TrFE), is a piezoelectric material, with a piezoelectric coefficient of 20 pC∙N^−1^. It is a biocompatible but non-biodegradable thermoplastic polymer, with high flexibility as well as chemical and physical resistance. It has been used in various tissue engineering studies and recently also for the regeneration of cardiovascular tissue [22,135]. The addition of piezoceramics such as ZnO nanoparticles and boron nitride nanotubes (BNNTs) can increase piezoelectricity and other properties of piezoelectric polymers. ZnO nanoparticles added to a P(VDF-TrFE) scaffold improved the adhesion and growth of HUVECs and MSCs seeded on the scaffold in vitro and stimulated angiogenesis in rats [24]. A porous scaffold was also created based on polyurethane and PDMS doped with barium titanate nanoparticles (BaTiO_3_), a highly biocompatible ceramic material with high piezoelectricity coefficient of 191 pC∙N^−1^ [136], to combine elastic with piezoelectric properties. The addition of piezoelectric nanoparticles improved the mechanical properties, making them comparable to the vessel ones. The scaffold in both presence and absence of nanoparticles was seeded with fibroblasts, that showed a better proliferation on the doped biomaterial counterpart (Figure 10) [137].

The mechanisms involved in controlling the aggregation of platelets on the vessel wall also seemed to be affected by the charge variations: electrical stimulation of an endothelial cell culture led to a strong secretion of the mediator prostaglandin I2, which varied with the electrical stimulus variation [138].

Although quite effective in regenerating the endothelium, TEVGs need a long and expensive in vitro culture process before implantation and an error in the differentiation process can lead to an immature endothelium, with consequent implant failure [137].

## 5. Piezoelectricity in Bone Tissue Regeneration

The structural components of the bone tissue are arranged according to precise patterns ranging from nan-o to macro-scale [139,140]. In particular, the fibrillar component plays a fundamental role in the bone mechanical strength and this is particularly evident after fractures, which represent one of the most frequent problems affecting bone tissue, caused by trauma (e.g., due to impact or fall), and favored by certain pathologies (e.g., osteoporosis) [141,142]. After fracture, a new, initially disorganized, fibrillar component is synthesized, which is later converted in parallel fibers giving increased structural rigidity [143]. Bone grafts are the most common solution for repairing a non-union, namely a fracture that does not heal over a certain time; however, important limitations to this procedure still remain, such as donor site morbidity, risk of infection transmission and scarce graft availability. In order to be functional, bone implant must allow the recruitment of osteoprogenitor cells that are able to proliferate, differentiate, produce new mineralized matrix, and remodel the bone. Furthermore, it is essential that the implant is soon vascularized [144]. The use of biomaterials, such as bioresorbable polymers, potentially allows some limits of traditional bone implants to be overcome, thus avoiding multiple surgical interventions and generating a 3D structure with a microenvironment stimulating genesis of new bone. However, rejection of synthetic implants and their osseointegration remain frequent issues to be solved [145,146]. To be considered suitable for bone tissue regeneration, scaffolds should possess the following characteristics: adequate mechanical strength capable of withstanding mechanical loads which bones are normally subjected to [147], high biocompatibility to avoid inflammation and rejection [148], and suitable porosity that allows deep colonization by cells and neovascularization (suggested to be in a few hundred microns but also containing pores < 20 µm) [143]. The biomaterial must then be osteoconductive to promote the migration of osteogenic cells into the scaffold, and osteoinductive to recruit and allow the commitment of stem cells and progenitors [149,150,151]. Osteoinductivity is not a merely chemical stimulus. It was demonstrated that material nanotopography can induce MPC-MSC transition as a sole factor; in particular, nanogratings, used as a cell culture substrate, were able to promote cell morphological polarization and stretching, which finally resulted in phenotype change towards the osteogenic lineage [152]. These results highlighted that some surfaces generate physical stimuli able to address the cells toward specific lineages, thus opening to the interesting possibility of producing nanopatterned biomaterials inherently able to induce stem cell differentiation, without the use of specific growth factors or cell culture media.

Finally, the piezoelectric properties of the scaffold could be considered relevant to impart physiological-like stimulation, including bone and vascular tissues [4]. Indeed, by mechano-electric signals, bone regulates many phenomena such as the healing of fractures, bone growth and remodeling [44,139,153,154,155]. Upon compressive loads, the mechanical stress generates a series of events including electrical signals by virtue of collagen fibers, specifically, collagen type I [8,44,153].

**Figure 11:**
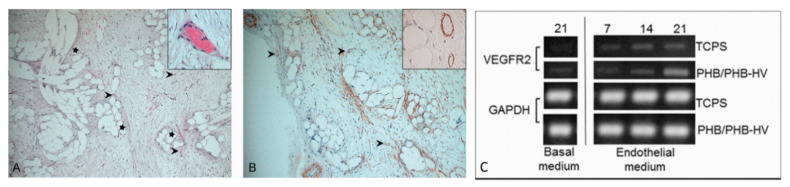
Vascularization in PHBHV-based fiber scaffolds: (**A**,**B**) PHBHV fibers after implantation in nude rats, arrows indicate the blood vessels, stars point to scaffold fibers [173]: (**A**) hematoxylin/eosin staining, and (**B**) 1A4 actin staining, reprinted and adapted under John Wiley and Sons Copyright Clearance Center (license number 5170801144911); and (**C**) PHB/PHBHV fibers cultured in vitro with adipose-derived MSCs: expression of VEGFR-2 as an endothelial marker [172], reused under Creative Commons Attribution License.

Realizing the piezoelectric nature of bone, in recent years several studies are being performed to evaluate the effects of piezoelectric polymers and nanoparticles on bone regeneration processes. Piezoelectric nanoparticulate systems, such as nanoceramics (i.e., BNTTs), mechanically activated via ultrasound after being up-taken by osteoblasts in vitro have proven capability of stimulating bone ECM formation by upregulation of TGF-β, a factor sensitive to electric signals [156]. On a macroscale level, PVDF was produced in different structures, such as films, membranes, and 3D scaffolds, tested in rat bone defects, where mechanical stress was provided by movement. After four weeks, PVDF films demonstrated BM and trabecular bone formation. Fiber meshes improved bone regeneration compared to the flat surface of the films, showing that the morphological structure of the material was also involved in the control of the bone regeneration [157]. This aspect was also evaluated in a study employing P(VDF-TrFE) scaffolds, fabricated with hexagonal or linear morphology in order to evaluate which shape actually influenced the proliferation and differentiation of pre-osteoblasts, without using differentiation factors. The results showed that only the scaffold with hexagonal morphology elements could give rise to bone cell differentiation [158]. The incorporation of nanoparticles can improve many properties of the scaffold also in relation to its biocompatibility. For example, it was observed that the addition of ZnO and BaTiO_3_ nanoparticles in electrospun PVDF scaffolds improved the piezoelectricity of the material and the adhesion, proliferation, and differentiation of human MSCs [24,159,160,161]. The use of BaTiO_3_ nanoparticles increased the tensile strength, the piezoelectric coefficient, and the bioactivity of the P(VDF-TrFE) composite scaffold improved osteogenesis in vivo [159,160]. Despite the excellent results obtained in bone regeneration, PVDF family members are non-biodegradable so the artificial scaffold cannot be fully substituted by natural tissues. Having available piezoelectric yet bioabsorbable scaffolds is an important goal for clinical applications. One possibility relies on the use of polyhydroxyalkanoates (PHAs), a family of largely biocompatible and bioresorbable biopolyesters synthesized by microorganisms under certain metabolic conditions, exhibiting, in some members, piezoelectric properties [162,163]. However, PHAs are more hydrophobic than natural polymers, and well-known polyhydroxybutyrate (PHB) is rigid and difficult to process [164,165,166,167]. It has been observed that despite their low osteoconductivity, these materials showed a better differentiation capacity than synthetic polyester counterparts [168,169,170]. In addition, PHAs are able to bind to ceramic materials such as hydroxyapatite or natural polymers, thereby improving their mechanical properties and affinity with the biological substrate. The addition of hydroxyapatite improved the mechanical properties and osteoconductivity of PHAs [171,172], while the addition of bioactive glasses stimulated, towards their degradation products, the osteoprogenitor cells [173]. Finally, the addition of natural polymers, such as gelatin and chitosan, improved the biological properties and elasticity of these materials [174,175]. Among the PHAs, PHB and poly(hydroxybutyrate-co-hydroxyvalerate) P(HBHV) (or PHBV), which add piezoelectric properties to improved processability, are the most largely studied members [176,177]. PHB and P(HBHV) have longer degradation times than other biocompatible polymers and a piezoelectric coefficient of approximately 1.3 pC∙N^−1^, similar to that reported for human bone. The piezoelectric effect in these polymers originates mainly from their crystalline properties, by which an external stress applied, induces the internal rotation of the dipoles in the crystalline phase, which ultimately gives rise to electrical polarization [150,178]. MSC-seeded PHB and P(HBHV) fibers have been shown to improve vascularization in engineered bone tissue [179], and PHB scaffolds coated with collagen type I and chondroitin sulphate promoted osteodifferentiation and vascularization (Figure 11) [180].

Along with microsurgery, cited in Section 4, the strategies to improve the vascularization of an implanted scaffold also include co-culture with cells capable of endothelial differentiation and the in vitro pre-vascularization of the construct [112]. The addition of factors promoting osteogenesis and angiogenesis could also represent a further strategy to obtain vascularized bone constructs. Studies performed in this direction showed that the by loading an osteogenic enzyme, ALP, on a mineralized PHB electrospun scaffold, adhesion and proliferation of osteoblasts greatly increased in vitro [181]. Another material with characteristics of biocompatibility, bioresorption, and piezoelectricity is the poly(L-lactic acid) (PLLA) [182,183]. The piezoelectric effect depends on the crystallinity and orientation of the polymer and is determined by the displacement of the double bond between carbon and oxygen in response to mechanical stress, which leads to the creation of a dipole moment and electric charge [184,185]. The piezoelectric coefficient of PLLA has been reported to be 1.58 pC∙N^−1^ [186]; however, similarly to PVDF and PHB, it could be improved by post-fabrication treatments, like poling or mechanical stretching. PLLA scaffolds containing apatite and collagen stimulated the metabolic activities of osteoblastic cells in vitro [187]. The addition of BaTiO_3_ nanoparticles to a PLLA scaffold, with electrical properties mimicking those of natural bone, generated an electroactive membrane able to improve the cranial defect without the aid of bone grafts [188].

Natural polymers show improved biocompatibility if compared to synthetic scaffolds, and are usually bioresorbable. Regeneration of bone defects in rats ameliorated when collagen fibers were implanted in the damaged area [189], and collagen-based scaffolds were able to enhance bone cell proliferation and differentiation [190,191]. In addition, collagen-derived proteins, such as gelatin and collagen peptides, can be blended with piezoelectric polyesters, such as PLLA, to produce spongy scaffolds, thereby demonstrating optimal hemocompatibility (i.e., absence of blood clotting) and capability to support bone stem cell niches in vitro [192,193].

**Figure 12:**
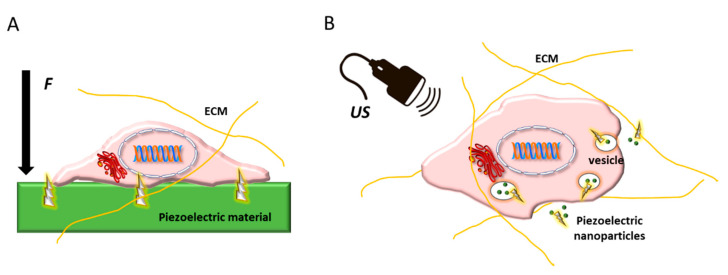
Schematic showing stem cell embedded in its extracellular matrix (ECM) and possible routes of using piezoelectric biomaterials to regulate its differentiation: (**A**) as substrates or scaffolds, which upon the application of an external force (*F*) give rise to electric stimuli in contact with the cell membrane; and (**B**) as nanoparticle systems trafficking intracellularly, which can be activated by ultrasound (US), thus inducing intracellular electric stimulation.

Among natural polymers, polysaccharides, such as chitin, chitosan, and cellulose, together with piezoelectric properties, can be beneficial in bone regeneration. Indeed, chitosan, derived from chitin, is a natural and antibacterial piezoelectric polymer exhibiting osteoconductive properties [194], and cellulose-based scaffolds significantly increases the osteoblast proliferation [195]. Due to the recognized importance of piezoelectricity in bone and vasculature to regulate differentiation of stem and progenitor cells, the use of piezoelectric materials as physically active substrates or as nanoparticles holds great promise for vascularized bone regeneration (Figure 12).

## 6. Conclusions and Future Perspectives

In Europe and the United States, more than half a million patients annually receive bone defect repairs with a cost estimate higher than 3 billion euros. Therefore, bone has become the second most transplanted tissue after blood. These numbers are globally increasing, due to a variety of factors, such as the growing needs of the world population, the increased life expectancy, and access to advanced health services and assistance. Arthroplasty revision surgery, oncologic surgery, bone fractures, non-union surgery, and otologic surgery, account for the most frequently graft-assisted reconstructive surgeries. Bone graft substitutes aim to provide the reconstructive surgeons with off-the-shelf alternatives to the natural bone taken from humans or animal species. Under the tissue engineering approach, new scaffolds can be designed incorporating bone stem cells to decrease the disadvantages of traditional tissue grafts via osteoconductive, osteoinductive, and osteogenic properties. The key steps towards optimized clinical application of tissue-engineered bone rely on neovascularization of the engineered construct. One of the major causes of poor implant integration and survival in standard engineered bone constructs is the lack of vascularization. The vascularization process is crucial in bone regeneration and healing as well as in remodeling and homeostasis, and the complex pathways that lead to angiogenesis and osteogenesis are interdependent. Without a good blood supply, the bone formation cannot begin and the implanted scaffold cannot be integrated in the host bone. Blood supply at the level of the bone defect is crucial to maintain the tissue viability in the initial phases after scaffold implantation and to allow a proper nutrient diffusion and waste removal. The development of a new generation of smart scaffolds that can promote bone regeneration and vascularization is one of the major challenges for bringing bone tissue engineering research into the clinical practice.

Having realized the special contribution of piezoelectric signals towards the differentiation and function of bone and endothelial cells, piezoelectric biomaterials, particularly those based on bioresorbable biopolymers, such as PHAs and PLLA, could ultimately permit the successful implant of bone substitutes through their effective vascularization in vivo. Piezoelectric, namely mechano-electric stimuli, can be sensed by stem cells. As intriguing examples, high output electric voltages can determine MSC osteogenic commitment, whereas the direction of an electric field vector has been able to induce endothelial and osteoblast alignment in a biomimetic fashion, revealing the prominent role of bioelectricity in bone. Many biological and some synthetic polymers possess appreciable piezoelectricity, whose demonstration has become the objective of fundamental studies over time. By mastering such physical cues, in terms of biomaterial and surface properties, including surface topography, it would be possible to enhance bone healing and regeneration capacity with significant clinical relevance. Despite the current lack of comprehensive studies designed to control the piezoelectric properties of scaffolds to regenerate vascularized bone as a whole, some in vivo studies are strongly suggestive of possible successful achievements led by piezoelectric stimulation.

## Data Availability

Not applicable.
